# The effect of online motivational interviewing on pregnant women's smoking cessation behaviour: A randomized controlled trial

**DOI:** 10.1111/ijn.13303

**Published:** 2024-10-04

**Authors:** Betul Esra Cevik, Semra Kocatas

**Affiliations:** ^1^ Susehri Health School Nursing Department Sivas Cumhuriyet University Sivas Turkey; ^2^ Faculty of Health Science, Department of Nursing Sivas Cumhuriyet University Sivas Turkey

**Keywords:** motivational interviewing, nursing, pregnant woman, smoking cessation, transtheoretical model

## Abstract

**Purpose:**

This study aims to examine the effect of online individual motivational interviewing based on the transtheoretical model on pregnant women's smoking cessation behaviour.

**Material and Method:**

Sixty‐two pregnant women who did not complete the 16th gestational week and who smoked were randomly assigned to intervention (*n* = 31) and control (*n* = 31) groups. While the intervention group was administered model‐based motivational individual counselling interventions, the control group was administered no interventions.

**Findings:**

The results showed that 58.1% of the pregnant women in the intervention group and 22.6% of the pregnant women in the control group ceased smoking.

**Conclusion:**

Online motivational interviewing based on the transtheoretical model was found to be effective in pregnant women's smoking cessation.

## Summary statement

What is already known about this topic?

- Smoking is one of the two leading causes of death worldwide.
- Smoking during pregnancy is a serious risk factor affecting both maternal and infant health.
- Nurses could make important contributions to raising non‐smoking generations and protecting and improving public health through trainings to be given to pregnant women.

What this paper adds?

- This paper reveals that online motivational interviewing based on the transtheoretical model was effective in pregnant women's smoking cessation.

The implications of this paper:

- The online motivational interviewing smoking cessation programme based on transtheoretical model, which forms the basis of our study, can be implemented by midwives and nurses without time, place and distance restrictions.

## 1 INTRODUCTION

Smoking is one of the two leading causes of death worldwide. There has been an increase in women's cigarette consumption as a result of women's increasing participation in work life, having economic freedom, moving away from social pressure and switching to a modern lifestyle and the use of all these targets by the tobacco industry (Bilir, 2022). The increase in cigarette use in the women population indicates an increase in the pregnancy‐related risks caused by smoking (Cinar et al., 2015). Studies conducted in Türkiye report pregnant women's smoking rates from 8% to 51.2% (Erbas et al., 2020; Kocak et al., 2015; Kocatas et al., 2020a; Kose, 2011; Marakoglu & Erdem, 2011; Tarhan & Yilmaz, 2016). Smoking during pregnancy is a serious risk factor affecting both maternal and infant health. Smoking during pregnancy has been reported to lead to many problems such as ectopic pregnancy, spontaneous abortion, placenta previa, detached placenta, ablasio placenta, premature rupture of membranes, pre‐eclampsia, preterm birth, stillbirth and congenital anomalies (Abide et al., 2018; Aktas & Guler, 2010; Izci & Bilici, 2015; Kilic, 2016; Moore et al., 2016; Neuman et al., 2012; Oztoprak & GuOnay, 2013; Pineles et al., 2016; Secen et al., 2017; Shobeiri & Jenabi, 2017; Sonmez, 2015; Tarhan & Yilmaz, 2016). Smoking during pregnancy has many negative effects on the foetus and early childhood period, which include foetal growth retardation, ~150–200 g decrease in birth weight and small head circumference (Abraham et al., 2017; Brand et al., 2019; Öztoprak, 2019), negative effects on foetal skeletal growth (Cutajar, 2022; Oztoprak, 2019), decrease in foetal lung capacity and alveolar count (Cinar et al., 2015), negative effects on the immune systems of foetuses and frequent respiratory tract infections in the infancy period, childhood asthma, cardiac disorders, otitis media (Keskinoglu & Aksakoglu, 2007), cleft palate/cleft lip, gastroschisis, atresia (anal or coanal), polydactyly, syndactyly, adactyly, bilateral renal agenesis and hypoplasia (Kilic, 2016) and serious problems such as cyanosis and absence of crying and breathing in the infant at birth (Kisacik & Golbasi, 2009).

Behavioural methods are safe methods that can be used for smoking cessation during pregnancy, so they are preferred more. Today, the frequency of the use of model‐based smoking cessation programmes is increasing due to their increase in the success of intervention. The transtheoretical model (TM) is the most frequently used model among these interventions. The use of internet, telephone and mobile application‐based interventions has recently increased in the treatment of smoking addiction (Korkmaz & Simsek, 2017). Online interviews enable people to express themselves openly and reduce embarrassment and stigmatization, so they are considered to be an effective method (Osilla et al., 2012). Online interviews conducted in familiar environments provide an opportunity for people to be more comfortable and enable more opportunities to reach people who are hard to reach (Pasaoglu, 2021). The use of internet‐based communication technologies is reported to be beneficial in increasing the applicability and effectiveness of motivational interviewing (Chan & So, 2021).

Determination of smoking rates in Turkey is difficult because studies are based on pregnant women's self‐reports. In addition, even if a pregnant smoker is detected, no effective smoking cessation method is applied, and pregnant women are generally recommended to reduce the number of cigarettes smoked, which is an insufficient approach for the creation of behavioural change in pregnant women. Nurses are recommended to continue their efforts to protect and improve the health of smoking pregnant women. Therefore, an online motivational interviewing smoking cessation programme based on TM for pregnant women is considered to be beneficial for pregnant women who are struggling to cease smoking, enable to reach more pregnant women at a lower cost by using the online interview method and enable pregnant women to complete the trainings without time, place and distance restrictions.

### 1.1 Hypotheses

Hypothesis 1a There is a difference between the intervention and control group pregnant women's smoking cessation rates after online motivational interviewing based on the TM.

Hypothesis 1b There is a difference between the intervention and control group pregnant women's Fagerström Nicotine Addiction Test scores after the online motivational interviewing based on the TM.

Hypothesis 1c There is a difference between the intervention and control group pregnant women's Self‐Efficiency–Efficacy Scale mean scores after the online motivational interviewing based on the TM.

Hypothesis 1d There is a difference between the intervention and control group pregnant women's Decision‐Making Balance Scale mean scores after the online motivational interviewing based on the TM.

Hypothesis 1e There is a difference between the intervention and control group pregnant women's Smoking Cessation Success Prediction Scale (SCSPS) mean scores after the online motivational interviewing based on the TM.

## 2 METHOD

### 2.1 Design

This study used a randomized controlled experimental design.

### 2.2 Sample size

This study was conducted in the family health centres of a city located in the Central Anatolia region of Turkey. The sample size of the study was determined using power analysis based on the study conducted by Balmumcu and Atan (2021) (G power 3.1.9.7, size: 0.76, alpha: 0.05, power: 0.90 and df: 1), and the sample size was determined as 60 pregnant women according to the analysis results. The power analysis was performed by a statistician. Considering any possible data loss, it was decided to include 66 pregnant women in the sample, 10% more than the calculated sample. Hence, the study included 33 pregnant women in the intervention group and 33 pregnant women in the control group. Inclusion criteria were being literate, being aged 18 years or older, not having completed the 16th gestational week, smoking a minimum of one cigarette a day and being able to use a smartphone or computer. Those who wanted to leave the study at any time and those whose pregnancy was terminated for any reason were excluded from the study.

### 2.3 Participants and randomization

**FIGURE 1:**
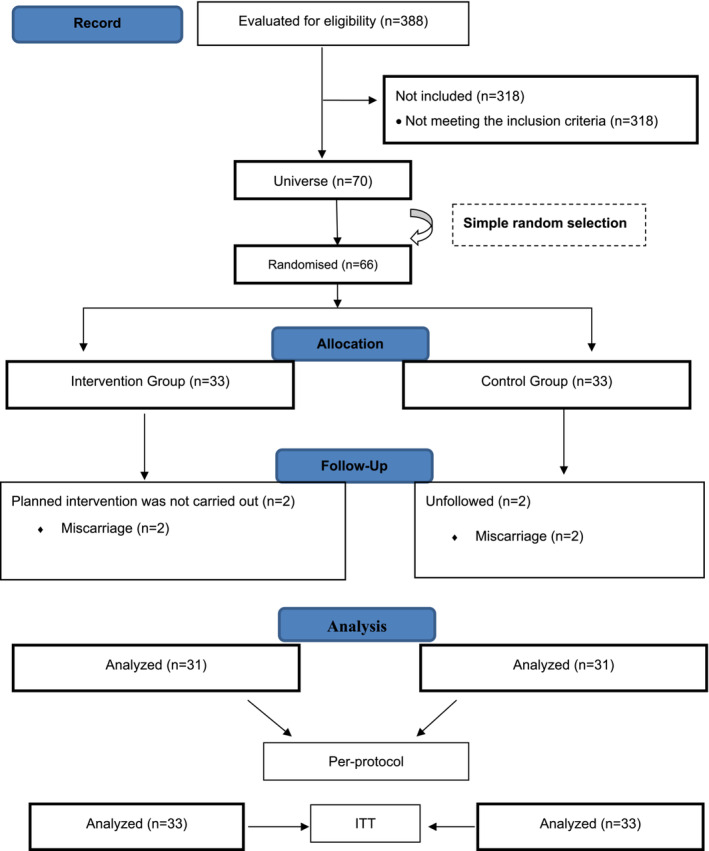
Consort of Research (2010) Follow Diagram

Sixty‐six pregnant women who met the inclusion criteria and agreed to participate in the study were divided into intervention and control groups by simple random sampling method. Two pregnant women from the intervention and control groups were excluded from the analysis due to miscarriage. The study was completed with a total of 62 pregnant women, 31 pregnant women in the intervention group and 31 pregnant women in the control group. The sample selection process was carried out in accordance with the criteria determined by the Consolidated Standards for Reporting Trials (Figure 1). Randomization was performed by a statistician other than the researcher. Randomization was performed after the pregnant woman's consent was obtained. In line with the sample size determined using power analysis, the group of the randomized controlled study (*n* = 66) was randomly selected from the population (*n* = 70) using a simple random numbers table. The pregnant women to be included in the intervention and control groups were listed by the statistician according to the randomization outputs. The intervention was performed by the researcher in this study, so the researcher was excluded from blinding. Blinding was performed in terms of statistical analysis and reporting, and the statistician who performed the data analysis was blinded. The research data were coded and transferred to the database as X and Y groups, without specifying which one was the intervention and control group. After the statistical analyses were performed by the statistician and the research report was written, the researcher explained the codings done for the intervention and control groups, which enabled the control of statistical bias, detection bias and reporting bias.

### 2.4 Data collection forms

#### 2.4.1 Pregnant woman information form

This form, which collected data about pregnant women's descriptive characteristics, was developed by the researcher in line with the literature (Balmumcu & Atan, 2021; Erbas et al., 2020; Karatay, 2007). The form includes 16 questions about women's sociodemographic characteristics, obstetric history and smoking history.

#### 2.4.2 Fagerstrom Nicotine Addiction Test

Fagerström (1978) developed a test to assess individuals' nicotine addiction levels. The Fagerstrom Nicotine Addiction Test was revised by Fagerstrom et al. (1992), and the Fagerstrom Nicotine Addiction Test was developed. A Turkish validity and reliability study was conducted by Uysal et al. (2004).

A total score of 0–3 points indicates a low level of addiction, 4–6 points indicate a medium level of addiction and 7 points and above indicate a high level of addiction. Cronbach's alpha coefficient of the Fagerström Nicotine Addiction Test is 0.61. Cronbach's alpha internal consistency coefficient was found 0.69 in this study.

#### 2.4.3 Stages of Behaviour Change Determination Questionnaire

This form consists of questions that determine the stages of thinking about smoking cessation. Respondents are asked to choose the appropriate option among the stages of change (Diclemente et al., 1991; Velicer et al., 1995). These stages include *pre‐thinking/not thinking* for those who responded “I do not plan to cease smoking in the next 6 months,” *thinking* for those who responded “I plan to cease smoking in the next 6 months,” *preparation* for those who responded “I plan to cease in the next 1 month,” *taking action* for those who responded “I cease less than 6 months ago” and *maintenance* for those who responded “I cease more than 6 months ago” (Diclemente et al., 1991; Greene et al., 1999; Velicer et al., 1995).

#### 2.4.4 Self‐Efficiency–Efficacy Level Scale

The scale, which was developed by Sherer et al. (1982) to evaluate behaviours and changes in behaviours, is a self‐assessment scale responded on a Likert‐type scale (1983). The scale was adapted into Turkish by Gozum and Aksayan (1999). Cronbach's alpha internal consistency coefficient of the scale was found to be 0.81. The scale consists of four subscales that include “initiation of behaviour,” “maintenance of behaviour,” “completion of behaviour” and “fighting obstacles.” Items 2, 4, 5, 6, 7, 10, 11, 12, 14, 16, 17, 18, 20 and 22 are scored reversely. Scores to be obtained from the scale range from 23 to 115, with higher total scores indicating higher self‐efficiency–efficacy perception and lower scores indicating low self‐efficiency–efficacy perception (Gözüm & Aksayan, 1999). Cronbach's alpha internal consistency coefficient was found to be 0.83 in our study.

#### 2.4.5 Decision‐Making Balance Scale

The scale developed by Velicer et al. (1985) was adapted into Turkish by Yalcinkaya and Karanci (2007). The scale was developed to reveal the benefits and harms of changing behaviour about smoking. The scale has two subscales, including “positive aspects of smoking” (1, 2, 5, 6, 7, 10, 11, 13, 19, 20, 21 and 24) and “negative aspects of smoking” (3, 4, 8, 9, 12, 14, 15, 16, 17, 18, 22 and 23). For both subscales, scores to be obtained from the scale range from 12 to 60. While perceiving the positive aspects of smoking indicates obstacles to behaviour changes, perceiving the negative aspects of smoking indicates positive behaviour change. The total score of the scale is obtained by subtracting the perceived harm of smoking total score from the perceived benefit of smoking total score. While a negative (−) result indicates that the perceived harms of smoking are dominant in the decision‐making balance, a positive (+) result indicates that the perceived benefits of smoking are dominant in the decision‐making balance. Cronbach's alpha internal consistency coefficient was found to be 0.82 in this study.

#### 2.4.6 Smoking Cessation Success Prediction Scale

The scale was developed by Aydemir et al. (2019), who also performed the validity and reliability of the scale. The SCSPS includes 10 items responded on a 5‐point Likert scale. The ninth item of the scale is scored reversely. The scale has two subscales, including “steadiness and readiness (1, 2, 6, 8, 9, 10)” and “health perception and appropriate environment (3, 4, 5, 7).” Cronbach's alpha coefficient of the scale was found to be 0.78. The scores to be obtained from the scale range from 10 to 50, with higher scores indicating a high level of smoking cessation success prediction. Cronbach's alpha internal consistency coefficient was found to be 0.84 in this study.

#### 2.4.7 Guide for motivational interviewing smoking cessation programme based on the TM in pregnant women

The guide was prepared by the researcher in line with the relevant literature (Balmumcu, 2019; Bhandari et al., 2018; Cengizoglu, 2019; Chaney & Sheriff, 2012; Claire et al., 2020; Crume, 2019; Cinar et al., 2015; García‐Gómez et al., 2019; Ioakeimidis et al., 2019; Karatay, 2007; Kocatas et al., 2020b; Koyun & Eroglu, 2013; Kutlu & Oksuz, 2018; Lindson et al., 2019; Ögel & Şimşek, 2021; Özel et al., 2019; Pineles et al., 2016; Scherman et al., 2018; Tarhan & Yilmaz, 2016; Tas et al. 2016; Toprak & Gülec, 2016) and used by the researcher during interviews with pregnant women. Views of eight experts from the faculty members in the departments of public health nursing and obstetrics and gynaecology nursing were consulted for the guide. The guide helped the researcher in determining and implementing the objectives and approaches appropriate to the stages of behaviour change.

### 2.5 Implementation of the study

The researcher went to the family health centres where the study would be conducted and determined smoking in pregnant women with a gestational week <16 weeks. The pregnant women to be included in the intervention and control groups were determined according to the randomization outputs. Pregnant women in the intervention group received motivational individual counselling training six times in accordance with the stages of change. Interviews were conducted online via Zoom.

The data of the intervention group were collected three times in total: before the training, in the 12th week and in the 6th month. The data of the control group were collected before the training and in the sixth month. After data were collected, an available day and time were determined with the pregnant women in the control group, and smoking cessation training was given to the pregnant women via Zoom. The study was completed between December 2021 and May 2022 with 31 pregnant women in the intervention group and 31 pregnant women in the control group.

### 2.6 Data analysis

Data were analysed using IBM SPSS V23. Normality distribution was analysed by Shapiro–Wilk and Kolmogorov–Smirnov tests. While an independent samples *t*‐test was used to compare normally distributed scores, a Mann–Whitney U test was used to compare nonnormally distributed scores. The comparison of the three normally distributed follow‐ups was done using repeated analysis of variance, and the comparison of three nonnormally distributed follow‐ups was done using the Friedman test. Multiple comparisons were performed using the Dunn test. The comparison of two normally distributed follow‐up scores was done using a paired sample *t*‐test, and the comparison of two nonnormally distributed follow‐up scores was done using the Wilcoxon test. The Pearson chi‐square test was used for the comparison of categorical variables, and multiple comparisons were performed using the *Z* test with Bonferroni correction. Analysis results were presented as frequency (percentage) for categorical variables and mean ± standard deviation and median (minimum–maximum) for quantitative variables. The significance level was taken as *p* < 0.05. Whether the losses experienced in our study were random was analysed using the Little's chi‐square test, indicating that the losses were totally random (Little's chi‐square = 468.387; *p* = 0.978). Missing data analysis was done using the EM method. To avoid bias, data were analysed by a statistician independent of the researchers. Analyses results obtained using per‐protocol (62 pregnant women) were included in the findings section of the study. The findings obtained by ITT analysis and per‐protocol analysis were similar.

### 2.7 Ethical considerations

For the implementation of the study, written permission was obtained from the Non‐Interventional Clinical Research Ethics Committee of Sivas Cumhuriyet University (Decision No: 2021‐06/16, Date: 23 June 2021) and the Provincial Health Directorate (Date: 30 November 2021, Number: E‐73192166‐044). Besides, a clinical trial number was obtained from ClinicalTrials.gov (NCT05173428). The researcher verbally informed pregnant women about the study and sent them the informed consent form via WhatsApp so that they could read it. The study included volunteer pregnant women and followed the principles of the Declaration of Helsinki.

## 3 FINDINGS

### 3.1 Participant characteristics

The average age of the pregnant women in the experimental and control groups was 27.72 years; 67.7% of the pregnant women had an educational level of high school or higher; 69.4% were employed; and 79% had a smoking spouse. Participating pregnant women were found to smoke an average of 11.48 cigarettes per day, and 82.3% had tried smoking cessation before. The groups demonstrated no statistically significant differences in terms of their sociodemographic characteristics (*p* > 0.05). Pregnant women in the intervention and control groups had similar sociodemographic characteristics (Table 1).

**TABLE 1:** Comparison of sociodemographic and smoking characteristics of pregnant women in the intervention and control groups.

Sociodemographic characteristics	Groups		Total	Test value	*p*
	Intervention group	Control group			
Age					
22–30	24 (77.4)	26 (83.9)	40 (61.3)	0.413	0.520^a^
31–38	7 (22.6)	5 (16.1)	12 (38.7)		
The average age					
Min–max (median)	22–28 (26)	22–38 (28)	22–38 (30)	418.5	0.380^b^
Mean ± *SD*	27.55 ± 4.09	27.9 ± 3.36	27.75 ± 3.72		
Education level					
Middle school and below	9 (29.1)	11 (35.5)	20 (32.3)	2.095	0.718^a^
High school and above	22 (70.9)	20 (64.5)	42 (67.7)		
Working or not					
Yes	21 (67.7)	22 (71)	43 (69.4)	0	1.000^c^
No	10 (32.3)	9 (29)	19 (30.6)		
Income level					
Income less than expenses	4 (12.9)	4 (12.9)	8 (12.9)	0	1.000^a^
Income equal to expenses	23 (74.2)	23 (74.2)	46 (74.2)		
Income more than expenses	4 (12.9)	4 (12.9)	8 (12.9)		
Family type					
Nuclear family	28 (90.3)	26 (83.9)	54 (87.1)	—	0.707^d^
Extended family	3 (9.7)	5 (16.1)	8 (12.9)		
Smoking features					
People who know you smoke					
My husband	6 (19.4)	8 (25.8)	14 (22.6)	6.186	0.289^a^
My family	5 (16.1)	7 (22.6)	12 (19.4)		
My friends	3 (9.7)	5 (16.1)	8 (12.9)		
Gynaecologist	6 (19.4)	3 (9.7)	9 (14.5)		
Midwife/nurse	4 (12.9)	0 (0)	4 (6.5)		
Nobody knows, I smoke secretly	7 (22.6)	8 (25.8)	15 (24.2)		
Your husband smokes					
Yes	26 (83.9)	23 (74.2)	49 (79)	0.389	0.533^c^
No	5 (16.1)	8 (25.8)	13 (21)		
Have you tried to smoking cessation					
Yes	25 (80.6)	26 (83.9)	51 (82.3)	0	1.000^c^
No	6 (19.4)	5 (16.1)	11 (17.7)		
Method used to smoking cessation					
I smoking cessation on my own	24 (92.3)	26 (100)	50 (96.2)	—	0.490^d^
I smoking cessation using medication	2 (7.7)	0 (0)	2 (3.8)		
Number of cigarettes smoked					
Min–max (median)	3–25 (11)	3–25 (12)	3–25 (12)	409.5	0.313^b^
Mean ± *SD*	11.06 ± 5.14	11.9 ± 4.19	11.48 ± 4.66		

^a^ Pearson chi‐square test.

^b^ Mann–Whitney U test, independent samples *t*‐test, mean ± standard deviation; minimum–maximum (median).

^c^ Yates correction.

^d^ Fisher's exact test; frequency (percentage).

### 3.2 Comparison of pregnant women's smoking cessation according to their last follow‐up

An analysis of the smoking cessation status of the pregnant women in the intervention and control groups in the last follow‐up showed that 54.8% of the pregnant women in the intervention group and 22.6% of the pregnant women in the control group ceased smoking. The groups demonstrated a statistically significant difference between their smoking cessation rates (*p* = 0.019) (Table 2).

**TABLE 2:** Comparison of smoking cessation status of pregnant women in the intervention and control groups according to the last follow‐up.

Smoking cessation	Groups		Test value	*p* ^a^
	Intervention group	Control group		
Yes	18 (58.1)	7 (22.6)	5.507	0.019
No	13 (41.9)	24 (77.4)		

^a^ Yates correction.

### 3.3 Fagerström Nicotine Addiction Test mean scores

In the first follow‐up, the groups demonstrated no significant differences in terms of their Fagerström Nicotine Addiction Test median scores (*p* = 0.507). The last follow‐up showed that the Fagerström Nicotine Addiction Test mean scores were higher in the control group, and this difference was statistically significant (*p* < 0.001) (Table 3).

**TABLE 3:** Fagerström Nicotine Dependency Test average scores of pregnant women in the intervention and control groups, according to follow‐ups.

	*n*	Intervention group		*n*	Control group		Test value	*p*
First follow‐up	31	4.06 ± 0.81	4 (3–6)	31	3.94 ± 0.81	4 (3–6)	U = 436.5	0.507
Interim follow‐up	15^a^	1.71 ± 0.47	2 (1–2)	—	—	—	—	—
Final follow‐up	13^a^	1.43 ± 0.65	1.5 (0–2)	24^a^	3.08 ± 0.88	3 (0–4)	U = 20.5	<0.001

*Note*: Mean ± standard deviation; median (minimum–maximum).

Abbreviation: U, Mann–Whitney U test.

^a^ FNBT was applied to pregnant women who continued to smoke. FNBT was not applied to pregnant women who quit smoking.

### 3.4 Self‐Efficiency–Efficacy Scale mean scores

When the groups were compared, no significant differences were found in terms of the initiation of behaviour and maintenance of behaviour subscale mean scores. While no statistically significant difference was found in the completion of behaviour first follow‐up subscales, the median score of the intervention group was higher than the control group in the last follow‐up, and the difference between the groups was significant (*p* = 0.018). While a significant difference was found between the first follow‐up subscale mean scores of fighting obstacles mean scores (*p* = 0.026), the intervention group's mean score was found to be higher than that of the control group. A significant difference was found between the last follow‐up mean scores of the fighting obstacles subscale, and the intervention group mean score was found to be higher than that of the control group. While in the first follow‐up, no significant difference was detected between the Self‐Efficiency–Efficacy Scale total mean scores of the groups, the total scale score of the intervention group was found to be higher than that of the control group in the last follow‐up, and the difference between the groups was statistically significant (*p* = 0.048) (Table 4).

**TABLE 4:** Self‐Efficacy Scale scores of pregnant women in the intervention and control groups according to follow‐ups.

	Group				Test value	*p*
	Intervention group		Control group			
Beginning the behaviour						
First follow‐up (month 0)	29.74 ± 4.13	30 (20–39)	28.84 ± 3.47	29 (20–35)	*t* = 0.932	0.355
Interim follow‐up (third month)	29.74 ± 4.17	30 (20–39)	—	—		
Final follow‐up (sixth month)	29.84 ± 4.03	30 (22–39)	28.97 ± 3.36	29 (20–35)	*t* = 0.925	0.359
Test value	F = 1.298		t* = −0.387			
*p*	0.276		0.702			
Maintaining the behaviour						
First follow‐up (month 0)	25.61 ± 3.72	27 (18–34)	24.32 ± 3.52	24 (18–34)	U = 373.000	0.126
Interim follow‐up (third month)	25.58 ± 3.76	27 (18–34)	—	—		
Final follow‐up (sixth month)	25.61 ± 3.69	27 (19–34)	24.58 ± 3.5	25 (18–34)	U = 393.500	0.215
Test value	$\chi^{2}$ = 1		*Z* = −0.736			
*p*	0.607		0.462			
Completing the behaviour						
First follow‐up (month 0)	17.55 ± 2.46	18 (14–22)a	17 ± 2.19	16 (14–21)	U = 418.000	0.371
Interim follow‐up (third month)	18.19 ± 2.12	18 (14–22)ab	—	—		
Final follow‐up (sixth month)	18.39 ± 2.06	20 (16–22)b	17.16 ± 2.16	17 (14–21)	U = 317.000	0.018
Test value	$\chi^{2}$ = 21.347		*Z* = −0.682			
*p*	<0.001		0.495			
Fighting obstacles						
First follow‐up (month 0)	11.29 ± 1.4	11 (9–14)	10.45 ± 1.36	10 (9–13)	U = 326.500	0.026
Interim follow‐up (third month)	11.42 ± 1.15	12 (9–14)	—	—		
Final follow‐up (sixth month)	11.42 ± 1.15	12 (9–14)	10.68 ± 1.33	11 (9–13)	U = 336.000	0.031
Test value	$\chi^{2}$ = 0.087		*Z* = −1.133			
*p*	0.957		0.257			
Total score						
First follow‐up (month 0)	84.19 ± 8.34	84 (72–106)	80.61 ± 7.46	79 (69–99)	U = 353.500	0.073
Interim follow‐up (third month)	84.94 ± 8.1	85 (72–105)	—	—		
Final follow‐up (sixth month)	85.26 ± 7.93	85 (72–105)	81.39 ± 6.97	80 (73–99)	U = 340.000	0.048
Test value	$\chi^{2}$ = 9.5		*Z* = −1.4			
*p*	0.051		0.161			

*Note*: Mean ± standard deviation; median (minimum–maximum); a‐b indicates that there is no difference between the scores of impressions with the same letter in each group.

Abbreviations: $\chi^{2}$, Friedman test; F, repeated analysis of variance; *t*, independent samples *t*‐test; t*, paired two sample *t*‐test; U, Mann–Whitney U test; *Z*, Wilcoxon test.

### 3.5 Decision‐Making Balance Scale mean scores

While in the first follow‐up, no statistically significant difference was detected in the perceiving the positive aspects of smoking subscale, in the last follow‐up, the median score of the intervention group was lower than the control group, and the difference between the groups was statistically significant. Compared with the control group, the level of perceiving the positive aspects of smoking was found to be lower in pregnant women who underwent motivational interviewing (*p* < 0.001). The median score of the intervention group was higher in perceiving the negative aspects of the smoking subscale in the first follow‐up, and the difference was statistically significant (*p* < 0.001). In the last follow‐up, the median score of the intervention group was higher than that of the control group, and the difference was statistically significant between the groups. The level of perceiving the negative aspects of smoking was found to be higher in the pregnant women in the intervention group than in the control group (*p* < 0.001). While in the first follow‐up, no significant difference was found between the Decision‐Making Balance Scale total mean scores of the groups, in the last follow‐up, the level of perception of the harms of smoking was found to be significantly higher in the intervention group than the control group (*p* < 0.001) (Table 5).

**TABLE 5:** Decision‐Making Balance Scale scores of pregnant women in the intervention and control groups according to follow‐ups.

	Group				Test value	*p*
	Intervention group		Control group			
Positive aspects of smoking						
First follow‐up (month 0)	30.74 ± 3.91	31 (22–38)b	30.68 ± 3.9	31 (22–38)	U = 471.500	0.899
Interim follow‐up (third month)	26.03 ± 1.14	26 (24–28)a	—	—		
Final follow‐up (sixth month)	25.16 ± 0.9	25 (24–27)a	27.1 ± 1.94	28 (22–30)	U = 178.500	<0.001
Test value	$\chi^{2}$ = 42.467		*Z* = −4.12			
*p*	<0.001		<0.001			
Negative aspects of smoking						
First follow‐up (month 0)	46.74 ± 2.68	47 (40–51)	43.84 ± 3.51	45 (32–49)	U = 224.000	<0.001
Interim follow‐up (third month)	47.42 ± 1.89	48 (42–51)	—	—		
Final follow‐up (sixth month)	48.06 ± 1.06	48 (46–50)	45.74 ± 1.46	46 (43–49)	U = 111.500	<0.001
Test value	$\chi^{2}$ = 15.05		*Z* = −3.245			
*p*	0.051		0.001			
Total score						
First follow‐up (month 0)	−16 ± 5.27	−15 (−27 to −7)b	−13.16 ± 5.1	−13 (−21–3)	U = 353.000	0.072
Interim follow‐up (third month)	−21.39 ± 2.51	−21 (−27 to −16)a				
Final follow‐up (sixth month)	−22.9 ± 1.33	−23 (−25 to −20)a	−18.65 ± 2.32	−19 (−23 to −15)	U = 53.000	<0.001
Test value	$\chi^{2}$ = 39.113		*Z* = −4.379			
*p*	<0.001		<0.001			

*Note*: Mean ± standard deviation; median (minimum–maximum); a‐b indicates that there is no difference between the scores of impressions with the same letter in each group. The positive aspects of smoking subdimension refers to perceiving the benefits of smoking, and the negative aspects of smoking subdimension refers to perceiving the harms of smoking.

Abbreviations: $\chi^{2}$, Friedman test; *t*, independent samples *t*‐test; U, Mann–Whitney U test; *Z*, Wilcoxon test.

### 3.6 SCSPS mean scores

When the groups were compared, the first follow‐up indicated no significant difference between the steadiness and readiness subscale median value scores and health perception and appropriate environment subscale scores, but in the last follow‐up, a statistically significant difference was found between the median scores, and the median score of the intervention group was higher than that of the control group (*p* < 0.001). A comparison of the total scores of the groups showed that there was a statistically significant difference between the median scores of the first follow‐up and the last follow‐up, and the median total score of the intervention group was higher than that of the control group (*p* < 0.001) (Table 6).

**TABLE 6:** Smoking Cessation Success Prediction Scale scores of pregnant women in the intervention and control groups according to follow‐ups.

	Group				Test value	*p*
	Intervention group		Control group			
Determination and readiness						
First follow‐up (month 0)	21.9 ± 2.62	22 (17–27)b	20.65 ± 2.71	21 (15–26)	U = 361.50	0.091
Interim follow‐up (third month)	24.71 ± 1.55	24 (21–28)a				
Final follow‐up (sixth month)	25.39 ± 1.2	25 (24–28)a	22.29 ± 2.45	23 (17–27)	U = 129.00	<0.001
Test value	$\chi^{2}$ = 52.17		*Z* = −3.429			
*p*	<0.001		0.001			
Health perception and suitable environment						
First follow‐up (month 0)	14.65 ± 2.12	14 (10–20)b	13.74 ± 2.02	14 (10–18)	U = 350.00	0.058
Interim follow‐up (third month)	15.97 ± 1.58	16 (14–20)a				
Final follow‐up (sixth month)	16.81 ± 1.17	16 (15–20)a	14.74 ± 2.02	14 (11–20)	U = 167.50	<0.001
Test value	$\chi^{2}$ = 39.077		*Z* = −2.68			
*p*	<0.001		0.007			
Total score						
First follow‐up (month 0)	36.55 ± 4.54	36 (28–47)b	34.39 ± 4.43	34 (26–44)	U = 338.00	0.044
Interim follow‐up (third month)	40.68 ± 2.95	40 (35–47)a				
Final follow‐up (sixth month)	42.19 ± 2.02	42 (40–47)a	37.03 ± 4.13	36 (29–47)	U = 138.00	<0.001
Test value	$\chi^{2}$ = 52.061		*Z* = −3.414			
*p*	<0.001		0.001			

*Note*: Mean ± standard deviation; median (minimum–maximum); a‐b indicates that there is no difference between the scores of impressions with the same letter in each group.

Abbreviations: $\chi^{2}$, Friedman test; *t*, independent samples *t*‐test; U, Mann–Whitney U test; *Z*, Wilcoxon test.

## 4 DISCUSSION

An analysis of participating pregnant women's smoking cessation status showed that 58.1% of the pregnant women in the intervention group and 22.6% of the pregnant women in the control group ceased smoking. Smoking cessation status rates of pregnant women demonstrated a statistically significant difference between the intervention and control groups (*p* = 0.019) (Table 2). Based on these findings, Hypothesis 1a was accepted. The literature shows that the smoking cessation rates range from 4% to 76.2% in pregnant women (Dias‐Damé et al., 2019; Dokuzcan et al., 2020; Karatay et al., 2010; Keten & Gölbaşı, 2013; Riaz, 2017). The findings of this study are in line with some studies in the literature. The difference in smoking cessation rates is considered to be caused by the differences in interventions. Implementing different interventions and motivational interviewing instead of a single intervention during smoking cessation is considered to be more effective in behaviour change. Because pregnancy is a very convenient period to cease smoking, women are more willing to change their smoking behaviours and cease smoking during this period.

Hypothesis 1b was accepted according to the Fagerström Nicotine Addiction Test scores of the pregnant women in our study. The decrease in the Fagerström Nicotine Addiction Test scores of both groups in the last follow‐up compared with the first follow‐up could be related to pregnant women's being more motivated to cease smoking compared with other groups as well as the higher level of attachment between the mother and the baby with the progress in the gestational week. Balmumcu and Atan (2021) investigated smoking cessation with motivational interviewing methods in pregnant women and reported similar results. Except for one study, the literature was found to include no studies concerning smoking cessation using motivational interviewing in pregnant women (Karatay, 2007). On the other hand, the literature includes studies on smoking cessation using motivational interviewing in different groups. The findings of these studies are similar to the findings in this study, and the Fagerström Nicotine Addiction Test scores of the motivational interviewing groups were significantly lower than that of the control groups (Dilek, 2019; Tas, 2015). In line with these findings, the use of motivational interviewing together with TTM is considered to be effective in changing behaviour, reducing smoking behaviour and decreasing nicotine addiction levels.

When the Self‐Efficiency–Efficacy Scale scores of the pregnant women in our study were examined, Hypothesis 1c was accepted. Studies conducted on different groups reported that motivational interviewing increased individuals' Self‐Efficiency–Efficacy scores (Dilek, 2019; Evcimen, 2018; Gungormus, 2012; Gwaltney et al., 2009; İncirkus, 2016; Kamisli et al., 2017; Ridger et al., 2014; Tas, 2015). When smoking is evaluated as a behavioural risk factor, motivational interviewing is considered to be effective in fighting obstacles and completing the behaviour in order not to restart risky habits in times of difficulty and supporting pregnant women to perform smoking cessation behaviour by increasing their self‐efficacy.

When the Decision‐Making Balance Scale scores of pregnant women in our study were analysed, Hypothesis 1d was accepted. The literature includes studies on smoking cessation using motivational interviewing in pregnant women and different groups. In line with the results in our study, these studies also found that perceived benefit scores decreased and perceived harm scores increased in the intervention groups administered motivational interviewing (Balmumcu, 2019; Dilek, 2019; Erol, 2013). The literature also reports a finding that is not similar to our study findings (Koyun & Eroglu, 2013). This difference is considered to be caused by different research groups. In line with these results, online interviews based on TTM and motivational interviewing seem to have a positive effect on pregnant women's decision‐making balance.

When the pregnant women's SCSPS scores were analysed according to the follow‐ups, Hypothesis 1e of the study was accepted. Another study in the literature with a different group that utilized motivational interviewing reported similar results; SCSPS total and subscale scores of the intervention group receiving motivational interviews were found to be higher than that of the control group (Pasaoglu, 2021). The success of smoking cessation increases with the increase in the SCSPS score. The findings of the study and the literature knowledge show that motivational interviewing is effective in increasing smokers' smoking cessation success prediction. Predicting pregnant women's smoking cessation success is an important guide for approaching pregnant women. Therefore, more research should be conducted to predict smoking cessation success in pregnant women and to evaluate their motivation for smoking cessation.

4.1. Strengths and Limitations of Study

The strength of the study is that it is a Randomized Controlled Trial with a high level of evidence. Besides, blinding was performed in terms of statistician and reporting. Data collection tools consisted of valid and reliable scales. The use of appropriate statistical methods and ITT analysis in data analysis are also the strength of the study. The limitation of this study is that the determination of the smoking status of the pregnant women participating in the study was based on pregnant women's self‐report.

## 5 CONCLUSION AND RECOMMENDATIONS

The results of this study show that TTM‐based online motivational interviewing is effective in pregnant women's smoking cessation and reducing nicotine addiction scores. Online smoking cessation programme including motivational interviewing based on the TM, which is the basis of our study, is recommended to be implemented by public health nurses trained in this subject without time, space and distance restrictions. This research model should be applied in a larger sample; pregnant women's smoking status should be followed up in the postpartum and breastfeeding periods; and ITT analyses should be performed to make comparisons.

## CONFLICT OF INTEREST STATEMENT

The authors declared no potential conflicts of interest with respect to the research, authorship and/or publication of this article. This study was not funded.

## AUTHORSHIP STATEMENT

**Betul Esra Cevik:** Conceptualization; methodology; formal analysis; writing—review and editing. **Semra Kocataş:** Methodology; formal analysis; writing—review and editing; investigation.

## Data Availability

Research data are not shared.
